# Comprehensive and translational pathobiology of COVID-19 based on cellular and molecular techniques

**DOI:** 10.1016/j.plabm.2025.e00497

**Published:** 2025-08-11

**Authors:** Ali Akbar Samadani, Sogand Vahidi, Kosar Babaei, Seyedeh Elham Norollahi, Kourosh Delpasand, Elaheh Asghari Gharakhyli

**Affiliations:** a Guilan Road Trauma Research Center, Trauma Institute, Guilan University of Medical Sciences, Rasht, Iran; b Medical Biology Research Center, Kermanshah University of Medical Sciences, Kermanshah, Iran; c Noncommunicable Diseases Research Center, Neyshabur University of Medical Sciences, Neyshabur, Iran; d Cancer Research Center and Department of Immunology, Semnan University of Medical Sciences, Semnan, Iran; e Department of Medical Ethics, Guilan University of Medical Sciences, Rasht, Iran; f Department of Microbiology, Gorgan Branch, Islamic Azad University, Gorgan, Iran

**Keywords:** COVID-19 virus, Laboratory techniques, Pathogenesis, Transmission, Diagnosis

## Abstract

The biggest health issue in the world right now is the COVID-19 pandemic. This outbreak has caused a lot more people to be hospitalized for pneumonia and serious health problems, leading to many deaths. This report talks about many studies that showed the causes and how common COVID-19 is, as well as how to diagnose it in clinics and labs, and how to prevent and control it. These studies are very important and directly related to COVID-19 to help manage the current public emergency. Many parts of this dangerous disease, like how it spreads, how to diagnose it, how it infects people, and how to treat it, are still not well understood. It's important that to prevent, diagnose, and treat COVID-19 well, we need research at the molecular and clinical levels, along with public health measures and medical treatments. Clearly, new treatments like mesenchymal stem cell therapy have shown great promise in this area. Here, we will talk about and show the advanced lab methods used to understand how COVID-19 spreads, how it is diagnosed, and how it can be treated.

## Graphical abstract

**Figure:**
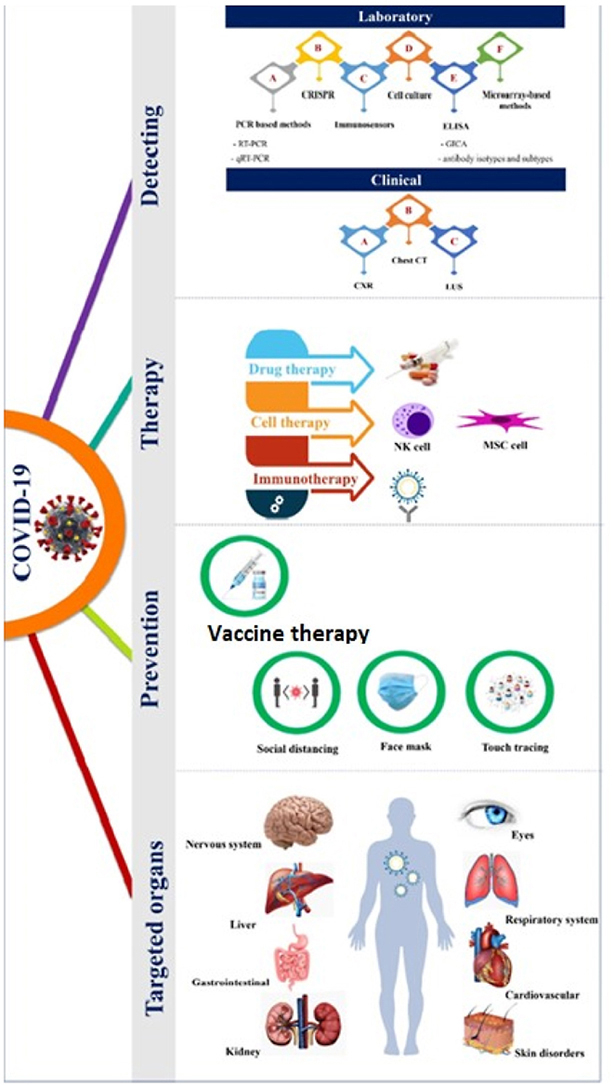

## 1 Introduction

**Fig. 1:**
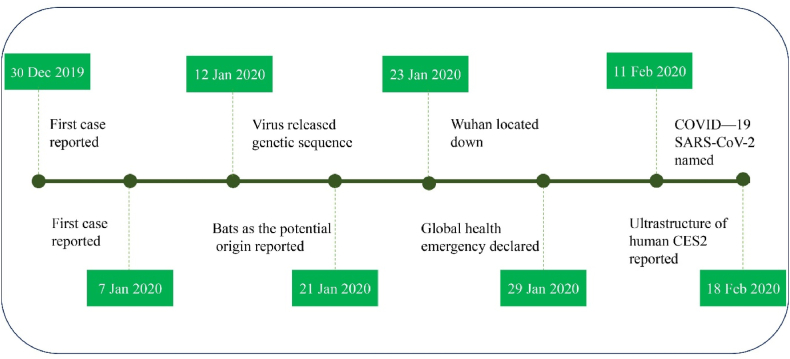
The epidemiology of COVID-19 from Dec 30, 2019 till Feb 18, 2020.

**Fig. 2:**
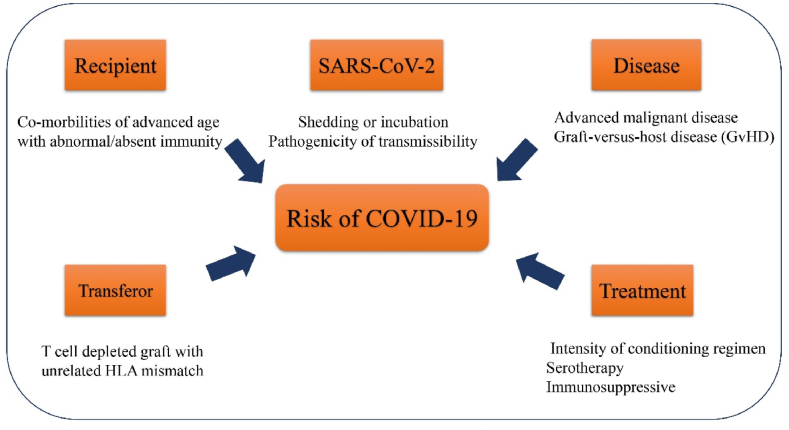
Overal association and effects of SARS-CoV2 and the risk of COVID-19. Other main elements which are involved in this category such as treatment, disease, recipient and transfer are indicated in this figure.

COVID-19 could be a clinical disorder caused by the Serious Intense Respiratory Disorder Coronavirus 2 or SARS-CoV-2 and is spreading quickly around the world [1] (Fig. 1). Doctors have noticed that being a man, being older, and having long-term health problems like heart disease or diabetes can raise the chances of getting COVID-19 and make the illness worse [2]. Luckily, very few children have died from the virus. SARS-CoV-2 is a type of virus that is 60–140 nm wide. It has four main parts: Spike, Envelope, Membrane, and Nucleocapsid. The Spike proteins look like a crown when viewed under a special microscope [3]. Four types of coronaviruses—OC43, HKU1, NL63, and E229—are found in people, and they usually cause mild cold-like symptoms. In the past twenty years, there have been two cases where a type of animal virus called beta coronavirus spread to humans and caused serious illness. The first case happened between 2002 and 2003 when a new type of virus from bats was passed to people in China's Guangdong Province through another animal. The virus called the acute respiratory syndrome coronavirus infected 8422 people in China and Hong Kong and caused the deaths of 916 people, which is an 11 % death rate, before it was brought under control [4]. Nearly ten years later, in 2012, the MERS-CoV virus appeared for the first time in Saudi Arabia. It came from bats and other animals and caused the deaths of 858 people, which is a 34 % death rate, after infecting 2494 people before it was controlled [5]. People of all ages can get the disease. The infection spreads through big drops of water in the air when a person who is sick or even someone who doesn't show symptoms sneeze or coughs. So far, there have been no reports of a mother passing an infection to her baby through the placenta. However, some cases of infection have been found after the baby was born [6]. The time it takes for the virus to show symptoms can be between 2 and 14 days. Some research says that the virus gets into cells in the lungs through a receptor called ACE2 [7]. COVID-19 can show a wide variety of symptoms. Some people may have no symptoms at all, while others may develop severe breathing problems. Common signs of illness are fever (not everyone has it), cough, sore throat, headache, tiredness and weakness, muscle pain, red or itchy eyes, and trouble breathing. So, it's hard to tell this virus apart from other things that can cause breathing problems. After the first week, some patients can get pneumonia, breathe badly, or even die. This process is linked to a big rise in certain proteins that cause inflammation, including MIP1A, MCP1, IP10, GCSF, IL10, IL7, IL2, and TNF-alpha [24]. Older people and those with existing health issues are more likely to experience side effects and die from COVID-19, making up about 50–75 % of COVID-19 deaths. Studies show that adult deaths range from 4 % to 11 %, while the overall death rate is estimated to be between 2 % and 3 % [8]. A study by Deeks and others. The goal was to accurately test for antibodies that detect COVID-19 infection. The study found a lot of variation in how people showed IgA antibodies. The presence of IgM and IgG antibodies, or combinations of them, changed at different times after symptoms started, ranging from 0 % to 100 % for all the targeted antibodies. The main findings of the study were from 38 other studies that sorted the results by how long the symptoms had been present. The overall results for IgG, IgM, IgA, total antibodies, and the IgG/IgM ratio were not very sensitive (less than 30. 1 %) in the first week after symptoms started. Sensitivity increased in the second week and was highest in the third week. The overall ability to detect IgG and IgM is 30. 1 % from 1 to 7 days, 72. 2 % from 8 to 14 days, and 91. 4 % from 15 to 21 days. The numbers in parentheses show the range of possible values for these percentages. Accuracy estimates for over three weeks were based on smaller numbers of samples and fewer studies. For 21–35 days after symptoms start, the tests for IgG and IgM have a sensitivity of 96. 0 % (with a confidence range from 90. 6 to 983)There isn't enough research to know how sensitive the tests are after 35 days. In 35 studies, the characteristics of all target antibodies were above 98 % with a confidence interval of more than 2. There were more false-positive results in people suspected of having COVID-19 but not confirmed, although these cases were few and the difference was expected to happen by chance. About 50 % of health care workers who think patients might have breathing problems expect that 43 out of every 1000 people will be tested for IgG/IgM between 15 and 21 days after they start showing symptoms. They won't be diagnosed, and 7 people (between the ages of 3 and 14) will get incorrect positive results. Based on a 20 % rate of occurrence in studies done in high-risk situations, if we test 1000 people, about 17 people (between 11 and 26) will not get diagnosed even though they should, and about 10 people (between 5 and 22) will be incorrectly told they have the condition when they do not. In a study where the condition is found in less than 5 % of people, about 4 out of every 1000 people tested will have the condition, while 12 out of 1000 will get a wrong positive result. In the end, it was found that antibody tests are not very useful for diagnosing COVID-19 in the first week after symptoms start. However, they might still help when used alongside other tests for people who get more severe illness later. When the RT-PCR test shows no result or hasn't been done. If antibody tests are done at least 15 days after symptoms start, they can help doctors find out if someone had a past infection with the SARS-CoV-2 virus (Fig. 2). We don't know how long the increase in antibodies lasts, and there isn't much information available for more than 35 days after the symptoms start. So, this study wasn't sure if these tests could be used for checking health in general. Worries about the high chance of bias and how it's used suggest that the accuracy of tests in medical care might be lower than what studies say. Sensitivity was mostly tested in patients who were in the hospital, so we don't know if these tests can find smaller amounts of antibodies that might be present in people with milder or no symptoms of COVID-19 [9]. In a study by Dong X and others, they looked into and explained coronary heart disease. We gathered electronic medical records from 11 COVID-19 patients. We looked at their background information, symptoms, important test results, and medical images. Symptoms can be mild, severe, or none at all, and some people may or may not have pneumonia. Testing in a lab for viral genetic material can sometimes give incorrect negative results. It's a good idea to also consider testing for specific antibodies (IgG and IgM) to help with the diagnosis. Patients with common allergies did not have specific symptoms or serious problems. The symptoms were worse for people who already had chronic lung disease or a secondary bacterial infection in their lungs [10]. A study by Mayara Lisboa Bastos and others looked into some things. To check how accurate blood tests are for diagnosing COVID-19 in patients. The sensitivity of the ELISA test for measuring IgG or IgM was 84. 3 %, with a confidence range from 95. 6 % to 909 %For LFIAs, the sensitivity was 66 %, with a range from 49. 3 % to 793 %For CLIAs, the sensitivity was 97. 8 %, with a range of 46. 2 % to 100 %Sensitivity was higher at least three weeks after symptoms started (going from 69. 9 %–989 %) than in the first week (which was from 13. 4 %–503 %)We need better studies to check how accurate COVID-19 blood tests are [11]. Another study focused on accurately identifying blood test results in patients with COVID-19. The sensitivity of the ICG test, which checks for both IgM and IgG antibodies, showed the following results in people confirmed by nucleic acid testing: 11. 1 % in the early stage (1–7 days after symptoms start), 92. 9 % in the middle stage (8–14 days after symptoms start), and 96. 8 % in the late stage (more than 15 days after symptoms start). The ability of ICG to find suspected nucleic acids was 43. 6 % Also, the relationship between the blood sample and the blood plasma had a kappa Cohen value of 0. 93, which means there is almost perfect agreement between the two types of samples. In summary, the ICG blood test is reliable and accurate for diagnosing COVID-19 and is a helpful tool in medical settings [12]. Many studies show that patients with severe COVID-19 can have either a mild or strong reaction called a cytokine storm, which is a leading cause of death. So, treating cytokine storms has become very important for helping patients who are seriously ill. Interleukin 6 (IL-6) is an important part of a condition called cytokine release syndrome (CRS). If we can stop the IL-6 signal from working, it might be a new way to treat seriously ill patients. Tocilizumab is a type of medicine that stops a specific signal in the body called the IL-6 signal. So, Tocilizumab is probably a helpful medicine for people with severe COVID-19 [13]. We don't fully understand how the virus spreads or the different ways it can spread. The most common ways people get infections are by breathing in droplets from a cough or sneeze, from being close to someone who is infected, and from touching the mouth, nose, or eyes [14]. Indirect transmission is also possible due to contaminated surfaces (metal, glass, and plastic) on which the virus may remain for several days and may act as a secondary source [15]. Unlike earlier types of coronavirus, people with COVID-19 who don't show symptoms can still spread the virus through the air and by touching objects without feeling sick or even before they get sick. This might help us understand why the disease spreads so easily [16]. Recent research has increasingly focused on advanced diagnostic and modeling approaches to better understand and mitigate the spread of COVID-19. Another study developed a deep learning-based framework using CT imaging to detect COVID-19 cases, which demonstrated high diagnostic accuracy and robustness in clinical scenarios [17]. Furthermore, fractional-order mathematical modeling has provided important insights into transmission dynamics and intervention effectiveness for COVID-19 [18]. Another study also introduced real-time estimation techniques using segmented epidemic models to dynamically track infection rates [19]. In addition, other studies have presented models that assess the impact of control measures and predict outbreak trajectories [20]. These recent contributions highlight the importance of combining biomedical knowledge with computational and mathematical tools to develop a more comprehensive understanding of this pandemic. The novelty of this study lies in its comprehensive, interdisciplinary review that integrates the molecular mechanisms of COVID-19 with clinical diagnostics and emerging cell-based therapies. Unlike previous works that address these areas in isolation, this manuscript presents a unified translational perspective, with a special focus on the therapeutic potential of mesenchymal stem cells (MSCs) in mitigating COVID-19-associated complications. This approach offers a unique contribution by bridging fundamental biology with evolving treatment strategies in a single framework.

### 1.1 Main types of diagnosing and treatment methods

Since the 2019-nCoV is a new virus, there are not many tests available to diagnose it. To find this virus in samples taken from patients (like mucus or a wash from the lungs), a very effective method called RT-PCR is used. It is suggested to collect samples from the lower part of the lungs, like spit, fluid from the lungs, and fluid from the windpipe. If we can't get a sample from the lower part of the lungs, getting a sample from the upper part of the lungs will still help. These samples are taken with swabs from the nose and from the mouth and throat. Collect samples within 24–72 h. Send them to the lab and keep them in the fridge at a temperature of 4–8 °C. If you can't send the samples on time, it is best to keep them frozen at −70 °C or colder until you send them [21].

### 1.2 Different kinds of vaccines and how they work

We need vaccines to help stop the spread of COVID-19 and to help us get back to the way life was before the pandemic. Many vaccine options are being created, and several have finished their final tests with good results [2]. Vaccines can be made using weakened or killed viruses, living cells, tiny particles, virus-like particles, special viruses that help carry something, pieces of genes, and proteins. All of these can be used to make vaccines [3]. There are several steps in the vaccine testing process. The vaccine is given to healthy volunteers first to check how well it works and what the right amount should be. After that, they check how well the treatment boosts the immune system and test how effective it is in a small group of healthy people. The next step is to check how well the vaccine works in preventing illness after it has been given to many people. Finally, after the vaccine gets approval, it is checked to see how well it works and what its long-term effects are [4]. Scientists used special immune cells called dendritic cells, which were changed with proteins from the SARS-CoV-2 virus, along with a boost of specific T cells from the immune system, to make the first vaccines [5]. Another vaccine was made using mRNA wrapped in tiny fat particles that contain instructions for making the spike protein. The vaccine was made using mRNA wrapped in tiny fat particles that also created a protein. Viral vaccines were also made using adenovirus 5 and chimpanzee adenovirus (called chAd), and they included the S protein [22,23] (Table 1). Examples include the Hib vaccine and the pneumococcal vaccine [24]. Each vaccine works differently depending on its type. Some antibodies help produce more antibodies, while others help activate T cells, or they can do both. All vaccines help the immune system work better, so the body can find and get rid of viruses or bacteria.

**Table 1:** The summary of drugs used for COVID-19 therapy.

Drug	Action method	Recommended doses	Treatment duration	Side effects
Ribavirin (Lopinavir/ritonavir, ribavirin and IFN-β combination)	Antiviral synthetic nucleosides	500 mg intravenous, twice/day or three times/day	Up to 10 days	Mood disturbances, GI disorders, extreme anemia and skin rashes
Remdesivir	Interferes with the polymerization of viral RNA.	200 mg intravenous for the first day and 200 mg intravenous for other days	10 days	GI disorders and hypotension
Tocilizumab	IL-6 receptor inhibitor	For people weighing ≥80 kg, 600 mg is injected 12 h apart. For people weighing <80 kg, 600 mg is injected first and 400 mg 12 h later. The final dose is injected 16–24 h later.	3 days	Hypertension, headache, Infections of the upper respiratory tract, transaminase elevation and rhinopharyngitis
Chloroquine/hydroxychloroquine	–	400 mg by mouth twice/day for the first day then by 200 mg twice a day	5–14 days	Anorexia, weight loss, fatigue, and diarrhea; elevated liver enzymes; hypoglycemia; and hearing loss
Arbidol	Inhibitor to viral membrane fusion	200 mg, three times/day	10 days	not considerable
Colchicine	Anti-inflammatory effects	0.5 mg twice/day	3 days	Tachycardia, diarrhea, myalgia, nausea,
Sarilumab	IL-6 receptor inhibitor	200–400 mg intravenous	Single dose	Infection of the upper respiratory tract, elevated transaminases, urinary infection, erythema at the injection site, neutropenia
Interferon β-1b	Immune modifier	250 mg every 48 h	14 days	Flu-like syndrome, myasthenia gravis, rash, nausea, diarrhea, lymphocytopenia, headache, and exhaustion
Interferon α -2b	Immune modifier	5 million units, inhaled twice/day	5–7 days	Flu-like syndrome, myasthenia gravis, rash, nausea, diarrhea, lymphocytopenia, headache, and exhaustion

### 1.3 DNA based vaccine

A DNA vaccine works by putting DNA pieces into the cells of a person or animal. These DNA pieces help the body make proteins that can trigger an immune response. This method helps activate the body's immune responses, which include both the use of antibodies and the actions of immune cells. The vaccine is made to deliver genetic material into the cell's nucleus in the host's body. If the vector gets to the right place, the mammal's promoter turns on, and the cell starts to read the gene that the vaccine uses. After producing transgenic (genetically modified) proteins, the antigen-presenting cells (APCs) show their MHC I and II protein pieces to the main T cells to start the immune response that helps fight off diseases. Plasmid DNA vaccines that contain the SARS-CoV-2 S protein have been made using mice and guinea pigs. Giving these vaccines in two doses helped protect rhesus macaques from COVID-19 by producing antibodies and specific immune cells called CD8^+^ T cells. In making COVID-19 vaccines, the goal is to create strong immune responses without causing problems [25,26]. Keep in mind that this immune response from cells can be activated and that the release of certain substances can also trigger responses from B-cells [6]. Plasmid DNA has parts that can trigger a natural immune response, like non-methylated CpG sequences. This helps boost the immune response to the antigens that are being expressed. Even though tests with human DNA vaccines show some immune responses, these responses are not strong enough to bring about important health benefits. DNA vaccines have advantages like creating strong and safe immune responses to germs [6,26].

### 1.4 mRNA based vaccines

mRNA vaccines are considered better than DNA vaccines because they don't need to go through a process in the host cell's nucleus. So, we can use a smaller amount without needing special delivery methods. Also, mRNA vaccines don't mix with the host cell's DNA and might have only clean viral proteins. Right now, RNA vaccines like mRNA-1273 and BNT162b2 work more than half the time. The S receptor binding part is made by a type of mRNA called BNT162b1, which is mixed with tiny fat particles. The BNT162b2 vaccine works better if more doses are given and helps keep the body's proteins stable. The mRNA-1273 vaccine also helps with steady blood flow. It encouraged CD4 + T cells to react and produce substances like IFN-, IL-2, and TNF- at different amounts [[7], [8], [9]]. The mRNA-1273 has been assessed by Moderna Inc. The SARS-CoV-2 S protein is coded in its steady shape. BNT162b1 and BNT162b2 are both utilized with another immunization created by Pfizer/Biotech. NT162b1 has been created as a lipid nanoparticular-formulated immunization for the nuclear-modified mRNA encoding trimerized SARS-COV-2 spike glycoprotein receptor-binding space (RBD) [10]. mRNA-based vaccines are a new type of vaccine that shows great promise compared to traditional ones. They are very effective, can be made quickly, and are inexpensive to produce while being safe to use [27,28]. An mRNA vaccine works by using a special molecule that tells our cells how to make a protein that helps our body recognize and fight germs. It helps the immune system by showing it what to look out for [29]. One of the best anti-mRNA medicines is anti-COVID-19 mRNA4, which helps stop COVID-19 infections. However, there have been some reports of unusual problems linked to the anti-Covid-19 treatment. 19 cases of myocarditis related to mRNA vaccines, mostly in young men and teenagers [30]. The US Centers for Disease Control and Prevention says that about 1 in 2 people get myocarditis or pericarditis. 6 patients for every million doses compared to mRNA5. In people aged 12–39 years. Even though it's not a common issue, anti-mRNA medicines work well in animals and humans. Many of these drugs have been created to help fight infections and various kinds of cancer. Anti-mRNA drugs are good at targeting specific tumor markers, and many clinical trials are underway. Right now, two mRNA vaccines for COVID-19 are allowed for emergency use: the Pfizer-BioNTech COVID-19 Vaccine and the Moderna COVID-19 Vaccine [31]. This vaccine works well to protect against COVID-19 virus [32]. There are many kinds of mRNA vaccines made to protect against infections and different types of cancer, which are used in research to see how well they work. In summary, mRNA vaccines are a strong option compared to traditional vaccines because they work well, can be made quickly, and are cheaper to produce. Even though there are some problems with the COVID-19 mRNA vaccine, these vaccines work well in animals and people. Different types of mRNA vaccines have been created to fight infections and different kinds of cancer. Unlike conventional antivirals such as remdesivir and hydroxychloroquine, whose efficacy has been questioned in large clinical trials, MSC-based therapies offer a host-modulating effect that targets the underlying inflammatory pathways—especially cytokine storms—which are often the critical cause of mortality in severe cases.

### 1.5 Protein/peptide-based vaccines

Protein S is a good target for making vaccines because it helps the virus attach to host cells and also boosts the production of antibodies that can fight the virus. A special peptide that helps show antigens, a part of the antigen that connects to a type of MHC class II molecule, and a chemical link between the invariant chain and the antigen part are all included in the process of making the combined peptide [11]. Generex company announced that they have made a COVID-19 vaccine using their special technology to boost the immune system. They are also making a part of the SARS-CoV-2 virus for tests on humans [12]. The NVX-CoV2373 is a COVID-19 vaccine being tested in clinical trials. It is made using a stable protein created with special nanoparticle technology from Novavax. You can see it from above. In clinical trials, neutralizing antibodies work well. The Novavax Matrix-M adjuvant works with NVX-CoV2373 to boost the production of neutralizing antibodies and strengthen the immune response [13]. Using the help of the immune system and a special comparison tool for genes, researchers tried to create a vaccine made of many pieces of protein to fight SARS-CoV-2, focusing on the envelope protein. Although we still need to confirm it through tests, this method helps make a new vaccine quickly [14]. A protein or peptide vaccine is a type of vaccine that uses pieces of proteins from germs to help strengthen the body's defense against diseases. Here are some research findings: The COVID-19 vaccines from Pfizer/BioNTech and Moderna use mRNA and have the best sequence to help the body make the S protein correctly and for a long time [33]. SARS-CoV and anti-mRNA drugs have shown promise in recent clinical trials with a new drug called −2. This progress is due to the use of lipid nanoparticles, which work well not just for the mRNA vaccine when injected into the muscle, but also when combined with other medicines and antibiotics. Different vaccine sites show a balance between how well they work in producing antibodies, how often T-cells are activated, side effects, and overall effectiveness. No single vaccine method has consistently shown the best results in all these areas. RNA-based methods have been very effective, but at higher doses, they can cause unwanted side effects and may need additional help to work best [34]. The immune response to the vaccine is also influenced by the ingredients added to it, like guiding specific types of T cells (Th1 or Th2). Many studies have looked at how well the COVID-19 vaccine works. COVID-19 vaccines that use mRNA are 94. 6 % effective, while those using adenoviral vectors are 80. 2 % effective, according to phase II/III clinical trials. Besides diarrhea and arthritis, the most common side effects of anti-mRNA drugs were reported. The aluminum adjuvant vaccine has fewer side effects in the body and at the injection site compared to other adjuvant and negative vaccines, except for some redness when it is injected [35].

### 1.6 Viral vaccines

The study used a special type of S. with five vectors made in a lab. In the adenovirus study, around 80 % of the people experienced mild to moderate side effects. In 60 to 70 percent of the people tested, there were good changes in their neutral antibodies and T cell response. Pre-existing antibodies that don't target specific carriers lower the body's antibody responses [36]. The coding for a vaccine that uses an adenovirus has been tested. This particular adenovirus does not reproduce, so it won't cause problems with the immune system when responding to the vaccine. In one case, healthy adults had enough immunity. The vaccine helps your body create T-cells and antibodies, which can fight off germs. After some participants got the boost, both groups showed an immune response and had more neutralizing antibodies [37]. In Russia, they are testing a mix of two types of adenoviruses, 5 and 26, both of which are S carriers. In the first stage, each vaccine was tested one by one. In the second stage, participants started with rAd26 and then moved on to rAd5. Some side effects included pain where the shot was given, feeling sleepy from hypnosis, headaches, sneezing, and knee pain. Both participants knew that there were high levels of IgG that blocked the binding of the S. receptor and also activated CD4^+^ and CD8^+^ immune cells. The safety and effectiveness of the vaccine in this study are similar to what previous research found about adenovirus vaccines, but there were some concerns about the quality of the results in this study [38,39]. A fully activated alum virus vaccine has also been tried out. Alum was given in different amounts, and the effects were checked. About 20 % of people who got the vaccine experienced side effects like pain and fever at the injection site, which was no more than what people in the control group experienced. The average amount of neutralizing antibodies was between 121 and 316 [40]. Pfizer/BioNTech BNT162B2, Moderna mRNA1273, and AstraZeneca ChAdOx1S are the first COVID-19 vaccines that were approved. Severe allergic reactions to the mRNA vaccines were reported soon after they were approved, but they were treated successfully [41]. Antibiotics help stop infections by making the immune system stronger. There are several kinds of antibodies, such as live antibodies, inactive antibodies, subunit antibodies, and viral antibodies [42]. Recent research has shown that new kinds of vaccines made with multiple pieces of viruses, called multi-epitope vaccines, are being developed. These use virus-like particles (VLPs) and viral nanoparticles to help protect against diseases and can also make current vaccines work better. Treating illnesses that don't have a good vaccine [42,43]. Recombinant viral vectors are also used to introduce foreign proteins to boost the immune system, providing protection similar to what is gained from a natural infection. Some examples of viruses are adenovirus, the virus that causes measles, and the smallpox virus. To create a vaccine vector, we need to ensure that it can stay stable in the genome, boost the immune system, is safe to use, and can be produced in large amounts [44]. Right now, there are many vaccines for different viruses, like the human papillomavirus (HPV) and SARS-CoV-2. The HPV vaccine helps protect against several types of cancers linked to HPV. Our HPV vaccines are approved to fight against harmful types of HPV and help protect you from it. The current vaccine is made using DNA technology to help create clean protein pieces called L1, which then come together to make empty shells of the HPV virus [43]. The SARS-CoV-2 virus has led to the creation of several vaccines, including the Pfizer-BioNTech (Comirnaty), Moderna, and AstraZeneca vaccines. More people are working to find safe alternatives to alcohol-based medications to make chemical products used in treatments and vaccines safer. Usually, these media are added with cleaned proteins, regular proteins, or broken-down proteins, which often come from animals or humans. The average for people without diabetes is better than the average for those with diabetes. However, using pesticides to study the disease still carries some risks. This means that we can use non-animal products instead of animals in medicine for better health. It is also a safe place. Cyclodextrins are also used to help treat and prevent diseases. They are used to help improve vaccines and to keep cells alive when frozen. They are also important parts of medicines that treat diseases [45]. Antibiotics are a useful medicine for preventing diseases. Recent progress in developing polyepitope vaccines has shown promise in making current vaccines better and in treating diseases that don't respond to antibodies. Antibiotics help the immune system to notice and fight off infections. Here are some key points about vaccine research. mRNA vaccine: The mRNA vaccine is a new kind of vaccine that uses pieces of the virus's genetic code to help the body build protection against the disease. They have been used successfully to make vaccines for COVID-19 and are being studied for other viruses [46].

Viral variants could make medicines that stop diseases less effective. Some research shows that certain types of the SARS-CoV-2 virus, which causes COVID-19, might not respond as well to vaccines in patients. Types of antibodies: There are several kinds of antibodies, such as weak antibodies, subunit antibodies, and anti-mRNA antibodies. Each type has its own pros and cons regarding safety, immunity, and cost [47]. Ways to deliver antibodies: There are different ways to deliver antibodies, such as using DNA tools, viruses, and modified viruses. These steps could impact how safe and effective the vaccine is. Antibiotics are very useful for treating infections. Current studies are looking into new ways to make and give vaccines to make them safer and more effective. Antibiotics help the immune system learn how to see and fight germs. They have the same harmful substances or proteins as disease-causing germs, which are created by killing or disabling the bacteria. This injection helps the body recognize germs quickly. It lowers the body's ability to fight infections, helps get rid of them, and ultimately keeps us safe from getting sick again later [48]. There are different types of antibodies, including weak antibodies, subunit antibodies, and anti-mRNA antibodies. Each type has its advantages and disadvantages regarding safety, protection, and cost [47]. Vaccines can be given in different ways, such as using DNA, viruses, or modified viruses. These methods can influence how safe, designed, and effective the vaccine is.

### 1.7 Molecular mechanism of COVID-19

**Fig. 3:**
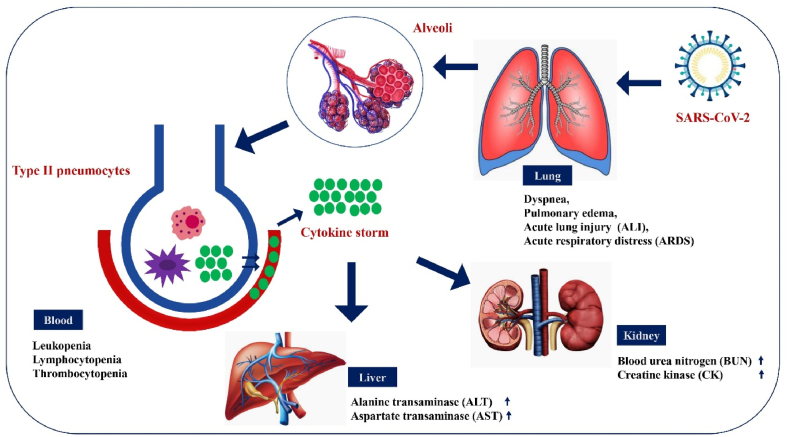
The pathologic effects of SARS-CoV2 on the targeted organs and their physiopathology involvement.

**Fig. 4:**
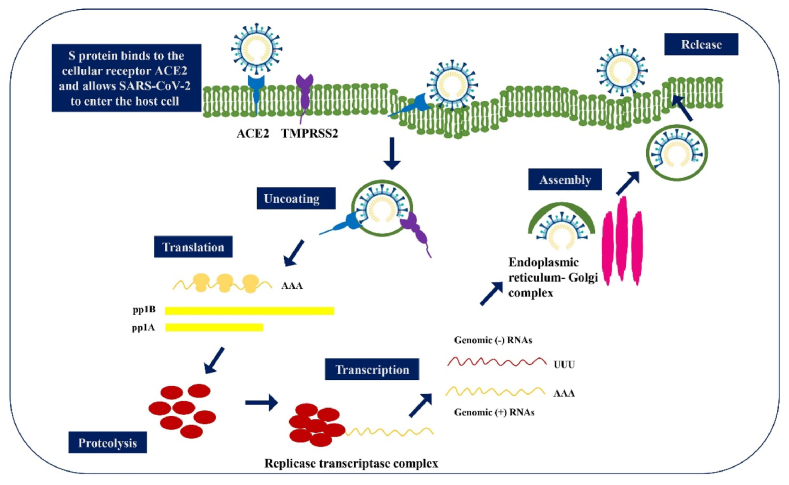
The pathobiology mechanism of COVID-19.

Coronaviruses are common germs that can make you sick in your lungs or stomach. Coronaviruses enter cells by using a special protein called S glycoprotein, which attaches to a specific part of the cell. Then, certain enzymes in the host cell help further with this process [49]. SARS-CoV-2 can attach to human cells better than SARS-CoV, which probably helps explain why SARS-CoV-2 spreads more often than other coronaviruses (Fig. 3). Unlike the non-ACE2 types, the new SARS-CoV-2 virus uses ACE2 to connect to and infect host cells. Many studies have shown that the SARS coronavirus can easily enter human cells through its connection with ACE2 [16]. Chemokine Ligand 2 is a small chemical that draws in immune cells, such as basophils, lymphocytes, and monocytes. Casein kinase II causes a change (phosphorylation) in the ACE2 receptor at a specific spot (Ser-787), and this process is influenced by damaged lung cells. SARS-CoV finds and connects to the ACE2 receptor, causing it to change shape. SARS-CoV gets into host cells by merging with the fatty layer of the cell's outer membrane. A key cut on the S protein at the S2 spot helps the virus and the host's cell membranes join together [50]. The viral RNA is changed into proteins inside the host cell's fluid. The Orfs that make Nsps are first changed into proteins called polyproteins (pp1a and pp1ab). These polyproteins are then cut into Nsps with the help of a virus-made enzyme called protease [49]. A specific group of smaller RNAs makes proteins that help build and support functions. S, M, and E are kept safe in the endoplasmic reticulum before they move to the Endoplasmic Reticulum-Golgi Intermediate Compartment (ERGIC). The viruses are put together at a part of the cell called ERGIC, and then they are released from the cell through a process called exocytosis [21,51] (Fig. 4). Different immune cells, like B and T lymphocytes, dendritic cells, natural killer (NK) cells, and macrophages, release proteins called cytokines. These cytokines play an important role in the body's response to inflammation [52]. With increased viral load, an uncommon host immune response occurs which leads to a continual activation and expansion of immune cells that generate enormous quantities of cytokines, In other words, a cytokine storm occurs (CS). The high levels of proinflammatory cytokines and chemokines including IL-1, IL-1β, IL-1R2, IL5RA, IL-6, IL, IL-10, IL-12, IL-18, IL-33, IFN-γ, TNF-α, TGFβ, CXCL1, CXCL2, CXCL8, CXCL17, CCL2, CCL3, CCL4, CCL5, CXCL8, CXCL9, CXCL10, CCR1, CXCR2 recognized in the lungs of COVID-19 patients [[53], [54], [55]]. Pro-inflammatory cytokines play a key role in the development of acute respiratory distress syndrome (ARDS) in patients with COVID-19 [1,56]. Patients with COVID-19 who developed ARDS had more IL-6 in their blood compared to those who did not have ARDS. People who died from COVID-19 and ARDS had higher levels of IL-6 in their blood compared to those who survived. Reducing the activity of the IL-6 signaling pathway might help prevent COVID-19. Different versions of a gene called human leukocyte antigen (HLA), such as HLA-B∗0703, HLA-Cw∗0801, HLA-B∗4601, and HLA-DRB1∗1202, are connected to how likely someone is to get infected by SARS-CoV [57].

### 1.8 Epigenetic involvement of COVID-19

**Fig. 5:**
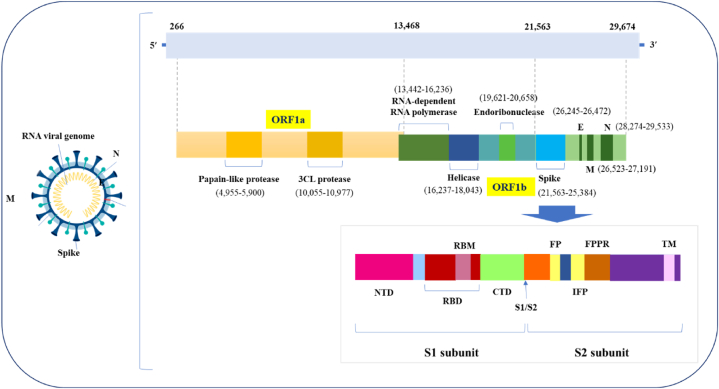
SARS-CoV-2 genomic characteristics.

Epigenetics is very important in causing and developing some common illnesses, especially those that happen as people get older. It also affects how likely people are to get different diseases, especially viral infections [58]. Different processes affect how the gene works. DNA methylation and changes to histones help reshape chromatin. The mix of DNA changes and changes to proteins, along with some types of RNA, helps make RNA and proteins in cells [59]. Changes made to chromatin, RNA, and DNA after translation, like adding methyl groups, acetyl groups, phosphate groups, or other small tags, play a role in how genes are turned on or off. By altering how a gene works without changing the DNA itself, this type of control links genetics (genotype) and physical traits (phenotype). DNA methylation (involving DNMT1, DNMT2, DNMT3A, DNMT3B, and DNMT3L) is a way that genes can be changed without altering their basic structure. It involves special proteins called DNA methyltransferases (DNMTs) that attach small chemical groups called methyl groups to a specific part of the DNA called cytosine. This process can be passed down from one generation to the next [60]. DNA methylation plays a role in how cells grow and age. It is involved in processes like turning off one X chromosome in females, marking genes for expression, and controlling repetitive DNA sequences. All of this helps keep our genetic material stable [61]. People infected with SARS-CoV-2, the virus that causes COVID-19, have shown changes in their genes. This suggests that treatments aimed at these genetic changes might be useful for helping with SARS-CoV-2 infections. Cells notice and react to viral infections as an important trigger. Many host cells go through big changes right after they get infected to try to stop or slow down the virus from making more copies of itself. This means both the body's natural defenses and learned immune responses are used, and in severe cases, cell death can happen. Viruses have often lived alongside their hosts, figuring out ways to avoid or fight back against the host's defenses. Changing the host cell to help the virus grow is one way this happens. It turns the infected cells into factories that make more viruses. The cell tries to fight off the virus, but the virus uses the cell to help itself. Epigenetic processes manage how antiviral genes and the host factors that the virus needs to reproduce are expressed. So, treatments that focus on epigenetic pathways might help in treating COVID-19. Epigenetic pathways increase the production of substances called cytokines that trigger a strong inflammatory response. To balance the dose of genes between males and females, one of the two X chromosomes in female mammals gets turned off during their development. Xist is made from a special area called the X-inactivation center in females. It covers the whole X chromosome and helps make the chromosome inactive by attracting proteins that change how DNA is packed, specifically two groups of proteins called PRC1 and PRC2. During the XCI phase, PRC1 adds a small chemical group to histone H2A at lysine 119, while PRC2 adds three methyl groups to histone H3 at lysine 27. These actions mainly help keep genes turned off and provide a middle level of genetic control. They also help continue the silent state that is started by Xist RNA [62]. The ACE2 gene was found on the Xq part of the chromosome. This area produces less of the gene and has more protein compared to other parts of the chromosome [63]. The parts Lys31 and Lys353 of the human SARS-CoV S1-CTD were found to stick closely to human ACE2. The host's genetic information can increase the risk of getting COVID-19, as shown by changes in DNA that affect the ACE2 gene. As people get older, the methylation at the cg085599149 CpG site near the ACE2 gene goes down [64]. DNA methylation and histone control ACE2, which explains why ACE2 is important in disease [65]. Most viruses in the corona and influenza groups can't alter the host's genetic code, but they can change how genes are expressed in the host. Recent studies have looked at how viruses use tools in our cells to help them start infections, spread, and stay in the body. Also, new technology has made it easier to study the changes in genetic markers across the entire genome. Many types of viruses can affect the immune system in different ways, and it's likely that SARS-CoV-2 does this too. Changes in the body's genes over time might affect how immune cells work, especially in fighting viruses. In terms of COVID-19, the connections between histone citrullination and the ability of stem cells to become different types of cells are important. Histone citrullination is important for stem cells to be able to become any type of cell. When the process of histone citrullination is stopped during the early development of embryos, there are fewer pluripotent cells [66]. The result of getting infected with SARS-CoV-2 is affected by different layers of gene control. The virus avoids the defenses of the host cell's immune system by changing the cell's functions. It creates a situation that helps the virus make copies of itself, put together new virus particles, and spread to infect more cells. COVID-19 has been hard to control because it spreads easily and can be passed on quickly before people show symptoms, making it tough to keep infected patients in quarantine. We need new ways and technology to fight this virus. Cytokine storms cause severe lung problems and a lot of damage to body tissues, which can lead to organ failure and death. The levels of IL-1, IL-6, IL-18, IFN-β, and TNF-α can change if we interfere with the body's genetic controls [67]. Treatment methods that focus on certain proteins related to gene control can help the infected patient. This can stop the virus from making copies of itself and spreading. To end the COVID-19 pandemic, treatments need to be provided to people around the world quickly and at a low cost. This will require teamwork from governments, drug companies, and others involved. We need to study the basic biology of SARS-CoV-2 and related viruses more to help stop the pandemic and make sure that any future viral outbreaks don't cause as much trouble. The decline in how mitochondria function is a key factor in age-related health problems linked to many diseases, including heart issues, brain disorders, cancer, and diabetes [68,69]. Problems with how the body processes energy and nutrients, like fat, sugar, and genetic material, are also connected to how severe and deadly COVID-19 can be [70]. The ongoing COVID-19 pandemic shows how important mitochondria are for the body's general immune response to viral infections [71]. COVID-19 prevents the body's immune system by not properly controlling a process called oxidative phosphorylation and by preventing a process that removes damaged mitochondria. This leads to the buildup of faulty and broken mitochondria in the cells [72]. Infection and immunity affect how mitochondria (the energy makers in our cells) work, how they produce energy, and how they change shape in different ways. Table 2 talks about how different bacteria and viruses affect mitochondria. Research on new vaccines came together to help create important vaccines [73]. Even with close checking of biologics, small-molecule treatments, and approved medicines, there has been no helpful antiviral treatment for COVID-19 so far [74]. Because germs need to get their nutrients from the host they infect, changes in SARS-CoV-2 can be an interesting sign to look out for [75]. A first report from a study shows that ongoing unusual low energy levels are connected to "long-term COVID" in people who have recovered from COVID-19 [76]. Some people with COVID-19, especially those with diabetes, may have small blood vessel problems in their eyes. High blood pressure and different types of diabetes are two of the most common things that can make COVID-19 more dangerous [77]. A study on school-aged children looked for the COVID-19 virus, SARS-CoV-2, and found that only a few children had a severe form of the disease. Most of them got better over time [78]. The irregular round particle, which is 70–90 nm wide, is present in SARS-CoV-2. Coronaviruses are types of viruses that have a single long strand of RNA, which is 30,000 letters long. This RNA is protected by special sections at the beginning and end [79]. The structure of SARS-CoV-2 is the same as most other beta coronaviruses. Small RNA sequences found in the SARS-CoV-2 virus show that they can reinsert themselves in specific random spots in the virus's genetic material. This suggests that coronaviruses have evolved over a very long period [80]. Six ORFs are produced by the genome and subgenome. ORF1a/b, which encodes sixteen nonstructural proteins, takes up the bulk of the 5′ end [81]. ORF1a/b makes a large protein that is cut by two enzymes, one in NSP3 and another called 3C-like protease. This process creates a group of proteins needed for the virus to copy itself and make its genetic material. The extra ORFs contain instructions for making possible helper proteins, along with Envelope-E, Spike-S, Nucleocapsid-N, and Membrane-M (Fig. 5). Table 3 presents the unique purpose and responsibility of every protein in the virus's life cycle. In comparison to SARS-CoV-1/SARS-like coronaviruses, there are 27 distinct amino acid replacements. SARS-CoV-2 has a greater infectivity and reduced cytotoxicity than SARS-like coronaviruses due to these changes [82]. SARS-CoV-2 has two main types, L and S, which are different because of two small changes in their genetic code. These are located in ORF1ab (T8517C, same meaning) and ORF8 (C251T, S84L) in the 8782 and 28114 parts. The L and S types of SARS-CoV-2 have small genetic changes that won't impact how our immune system responds [83]. The main parts of the SARS-CoV-2 virus are called S, N, E, and M proteins. S is a large protein that spans across cell membranes and has many roles. It is made up of two parts called S1 and S2. N is the only protein that connects to the virus's RNA, helping to put the virus together and improving how well the virus makes copies of itself. The structural protein E is the least important structural protein. It works like a tiny hole in the virus and is needed for several steps in the virus's life cycle, including causing illness, getting out of cells, and putting itself together. Matrix glycoprotein is the main viral protein that helps shape the viral membrane and is necessary for the virus to grow and put itself together [84]. Epigenetics deals with various mechanisms that cause long-term effects on gene expression patterns without changing the sequence of nucleotides. Over the years, epigenetics has led to great advances in biology, oncology, immunology, and the study of disease and infections. Coronavirus is one of the infectious diseases known to cause respiratory diseases such as pneumonia and cough and can cause diarrhea and respiratory problems in animals. This disease can be transmitted from person to person or from person to animal through droplets. The membrane is translocated to the host cell by ACE-2 exopeptidase.

**Table 2:** Infectious factors consequences of mitochondrial process.

Infectious factors	Mitochondrial metabolism	mitochondrial pathway of apoptosis	Mitochondrial fragmentation causing mitochondrial actives
severe acute respiratory syndrome (SARS)	Reduction of DRP1 causes mitochondrial segmentation.		
Hepatitis C	Fatty acid oxidation is disrupted.		
Influenza A		Cell death increased.	Reduction of mitochondrial membrane potential causes fragmentation.
Human immunodeficiency virus (HIV)		Apoptosis	Reduction of DRP1 causes mitochondrial segmentation.
Helicobacter	Improved aerobic glycolysis		Mitochondrial fragmentation caused by VacA, cytochrome *c* release into the cytosol, and death of cells
Zika virus		Cell death stopped	
Legionella	Improved aerobic glycolysis	Cell death stopped to raise intracellular proliferation of bacteria	Mitochondrial segmentation caused by DRP1
Herpes	Improved mitochondrial respiration and yielded TCA cycle		
Shigella		Apoptosis	Mitochondrial segmentation caused by DRP1
Listeria			Mitochondrial segmentation caused by DRP1
Dengue			Mitochondrial fragmentation occurs when DRP1 is suppressed.
Brucella			Lactate generation through aerobic glycolysis, TCA cycle inhibition

**Table 3:** SARS-CoV-2 nonstructural and structural proteins role.

Proteins	Roles
S	Linkage and viral entrance into the host cell are mediated by this protein.
N	It improves the virus's transcription rate and aids virion assembly.
E	It functions as a viroporin and is required for many phases of the virus cycle, including pathogenic assembly and viral releasing.
M	It is required for the morphogenesis and assembly of viruses.
ORF6	Reduction of IFN expression and effective in viral pathogenesis stimulation of apoptosis
ORF8	It appears to increase replication and bind with some structural proteins. Interacts with a few NSPs and an interferon antagonist.
ORF10	Its function is not clearly understood but may have an immune modulatory role Unknown (consists of 73 amino acid residues)
ORF14	Unspecified
ORF3a	It has the potential to boost replication and link with certain structural proteins. Interacts with some NSPs and an interferon antagonist.
ORF3b	Promoter of apoptosis
ORF7a	Activation of apoptosis
ORF7b	Unspecified
ORF9b	Demonstrate attraction with certain NSP
NSP9	Link to helicase
NSP16	5′-terminal methylated capped
NSP4	Viral replication-transcription complex and membrane reorganization
NSP6	Autophagosomes are formed as a result
NSP10	As either a methyltransferase activator, it modifies NSP16.
NSP11	Unspecified
NSP5	NSP polyprotein binds at numerous different locations
NSP7	A component of the replication-transcription complex, and builds complex with NSP8 and 12
NSP8	A component of replication-transcription complex that includes a heterodimer with NSP8 and 12.
NSP2	Suppression of the host translation mechanism as well as the innate immune system linkes to PHBs 1 and 2 and is thought to play a function in the triggering of apoptosis.

In conclusion, hypomethylation of angiotensin II converting enzyme (ACE II) may increase the risk of SARS-CoV-2 by improving its expression. Epigenetics refers to the modification of genes without changes in the DNA sequence. Recent research has explored the relationship between COVID-19 and changes in the cells of the cells [85]. A study looked at how sodium butyrate (TABIS) affects acute respiratory distress syndrome (ARDS) caused by staphylococcal enterotoxin B (SEB). The study found that drugs that affect gene regulation can make it easier and better to diagnose and prevent COVID-19-related diseases. This method is helpful and can be used during the pandemic because it has a consistent group to study, doesn't require special genetic tests, and there are screening tools for the immune system available. It prevents serious diseases related to COVID-19. The system that deals with cannabinoids (a type of chemical in cannabis) is linked to behaviors related to anxiety and depression caused by stress from nicotine. This study supports recent findings that changes in how our genes are controlled, like histone acetylation, are linked to problems like anxiety and depression [86].

### 1.9 Pathobiology and therapy process of SARS-CoV-2

**Fig. 6:**
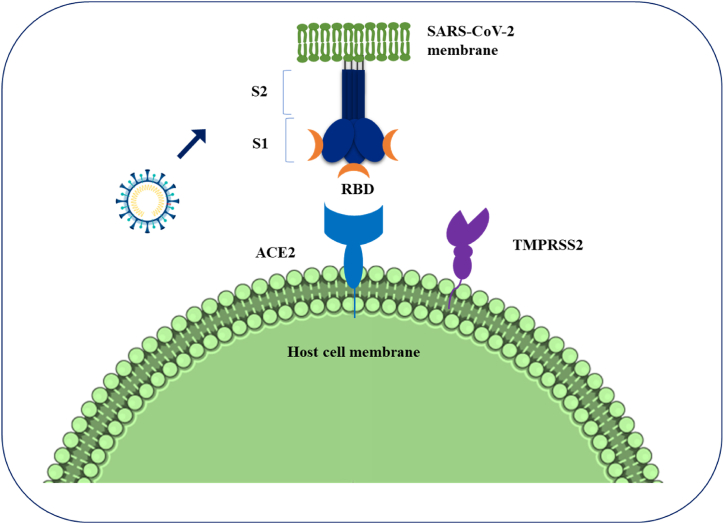
Coronaviruses' S protein aids viral entrance into target cells. Following virus assembly, the S protein of SARS-CoV-2 interacts to ACE2 as the entrance receptor via its S2 subunit. The S protein is destroyed at the S1/S2 limit or within the S1 subunit by TMPRSSs, which are cellular serine proteases.

**Fig. 7:**
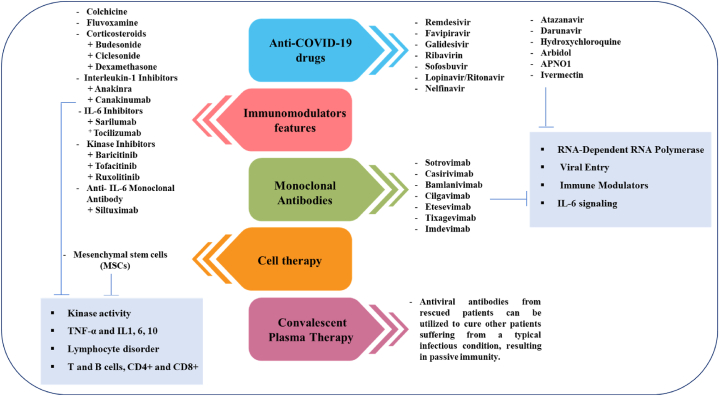
Significant and crucial facts for treatments programs that have been accepted or are in the development phase. COVID-19 diseases can be cured at many phases, including attachment, entrance, replication, and hyperinflammation.

The first SARS-CoV-2 infection happens when the virus attaches to human cells, like those in the nose and lungs. It connects to a protein called ACE2, which is found in many types of cells and contains zinc [87]. The virus enters the cell when the part of the virus called the receptor-binding domain (RBD) connects to a protein on the cell's surface called ACE2 [88]. The RBD of the S1 subunit links to the RBD of the ACE2, and the S2 component is in charge of membrane fusion (Fig. 6). Different helper proteins and activators, such as transmembrane serine protease 2 and certain enzymes found in cells called cathepsin B and L, help with loading the virus's S protein. These effects can cause problems with ACE2, which can break down and be removed from the cell. This leads to the shrinking of the tiny hair-like structures called cilia and a change in the type of cells in the lungs. Both of these issues can result in severe lung infections. [89]. SARS-CoV-2 could get into target cells by connecting to a new receptor called CD147. When the production of the CD147 protein is reduced, infection of cells by the new coronavirus goes down by 50 % [90]. Possible medicines for SARS-CoV-2 might work by stopping the virus from making copies of itself, blocking certain proteins that help the virus grow, or changing how the body's inflammation response works. Fig. 7 shows an example of treatment options that are available and those that are still being developed. It includes information about the type of medicine, the name of the product, the stage of medical testing, the company that makes it, how it works, the amount to take, and any drawbacks [91]. Lower viral levels were found in COVID-19 patients who were given medicines that stop the SARS-CoV-2 virus from connecting with human cells [92]. There are different treatments available, including antiviral medicines like ivermectin, special antibodies called neutralizing monoclonal antibodies (bamlanivimab and etesevimab), other types of human antibodies (casirivimab, imdevimab, and sotrovimab), and plasma from recovered patients. Many studies looked at how well ivermectin and hydroxychloroquine, either alone or with azithromycin, worked against the virus that caused COVID-19 at the beginning of the pandemic. Recent data shows that these medications might not reduce the risk of death or the time on a breathing machine, and they could possibly cause serious side effects [93]. The blood plasma or serum from people who have recovered from COVID-19 could help prevent and treat the infection in current COVID-19 patients, especially after they show symptoms [94]. The antibody connects to the S protein, which prevents SARS-CoV-2 from getting into the host cell and stops the virus from working. Also, the antibody changes the body's response to inflammation, and it's easier to do this in the early stages of the immune response, which usually doesn't show any symptoms. The finding that the human antibody CR3022, which is aimed at SARS-CoV, is connected to the part of SARS-CoV-2 shows that it could be a good treatment for SARS-CoV-2. It can be used by itself or with other strong medicines [95]. The ACE2/SARS-CoV-2 complex enters the cell using a special process called receptor-mediated endocytosis. Inside the cell, the virus loses its outer layer in acidic vesicles, which releases the virus's single-stranded RNA [96]. The host cell puts together the tools needed for the virus to make copies of itself and produce other important proteins from its single-stranded RNA. These proteins include special ones called replicase polyproteins and others labeled nsp1 to nsp16 [97]. When SARS-CoV-2 makes copies of itself, it creates its genetic material and many smaller versions. During this process, a change in the reading frame of the genetic code can happen. The coming together of viral RNA and proteins at certain parts of the cell called the endoplasmic reticulum and Golgi complex leads to the creation of virus parts [98]. In the end, these viruses leave the cells using a process called exocytosis. Many antiviral drugs have been shown to change how viruses make copies of themselves. They can directly target important viral proteins, like RdRp and viral protease, and can also disrupt how viruses use parts of our cells to reproduce. The way remdesivir, favipiravir, ribavirin, sofosbuvir, and tenofovir affect RdRp resulted in less production of viral RNA and mRNA binding [99]. Remdesivir is a promising drug that can fight many different human viruses, such as Ebola and various types of coronaviruses. It works by stopping the production of viral RNA. Also, other medicines like ritonavir, lopinavir, and darunavir have been looked at for people with COVID-19. These medicines stop a virus's helper enzyme from working, which keeps the virus from breaking down its proteins. Remdesivir appears to be the best antiviral medicine approved by the FDA for treating COVID-19 [100].

### 1.10 Inflammation reaction

The body's response to inflammation happens when a virus makes copies in lung cells. This response involves different immune cells like macrophages, antibodies, CD4^+^ T cells, cytotoxic T cells, and natural killer cells [101]. The epithelial-endothelial membrane is damaged during infection, which can lead to lung damage and problems in other parts of the body caused by SARS-CoV-2. Different treatments change how the body reacts to inflammation caused by SARS-CoV-2 in different ways.

### 1.11 Transmission of COVID-19 virus

The Huanan Seafood Wholesale Market was thought to be linked to the first spread of the SARS-CoV-2 virus in Wuhan in December 2019, and it was suggested that this market caused the outbreak. The disease spreads later from person to person. The way SARS-CoV-2 spreads easily may be because of its unusual features. SARS-CoV spreads most easily after a person gets sick and is highest when the illness is at its worst. In the first week of having symptoms, the amount of SARS-CoV-2 virus in samples from the upper respiratory tract was at its highest. This means there was a big chance of spreading the virus from the throat. SARS-CoV-2 spreads mainly through tiny droplets when people talk or are near each other. It can also spread a bit by touching dirty surfaces. We know that aerosol spread can happen, but we don't know how it affects people. People who carry the virus without showing symptoms are believed to be responsible for about 48–62 percent of the spread of the disease. It's hard to know how important it is for SARS-CoV-2 to spread from surfaces without knowing the smallest amount of virus needed to start an infection. Viruses can stay for a longer time on hard surfaces like stainless steel and plastic compared to soft ones like cardboard. The amount of virus on surfaces is believed to decrease quickly within 48–72 h. The finding that viruses can survive on surfaces shows that doorknobs, utensils, and clothes can carry SARS-CoV-2 and highlights the need for cleaning. However, the most common way the virus spreads is still through close contact between people. The amount of virus in the upper part of the respiratory system usually reaches its highest level around the time symptoms start. The virus can also be spread two to three days before symptoms show up. People who don't show any symptoms can still spread SARS-CoV-2 to others. In Singapore, there have been cases where people spread the virus to others up to 1–3 days before showing any symptoms themselves.

### 1.12 Diagnosis of COVID-19 virus

The amount of virus changes depending on the sample type, and samples from blood, breath, and stool can have very different amounts of the virus. In the first week of the illness, the amount of virus is very high and it reaches its highest point in the second week. The virus stays in the throat and anal swab samples for a long time (Table 4).

**Table 4:** Different types of diagnostic methods in COVID-19.

LABORATORY TEST	Antibody-based immunoassay	Antigen based immunoassay	Nucleic acid amplification test	Clinical tests
Detection methods	ELISA recognizing. Anti-SARS-CoV-2 antibodies, IgM or IgG	ELISA recognizing viral protein	Detection in genetic sequences of conserved regions for regions of the virus using RT-PCR and NGS	Clinical symptoms
Materials	Serum	Sputum, nasopharyngeal swabs, blood, endotracheal aspirate, bronchoalveolar lavage, stool	Sputum, nasopharyngeal swabs, blood, endotracheal aspirate, bronchoalveolar lavage, stool	Radiological test
Positive test results	IgM+ = days after onset, IgG = previous infection	Confirms the existence of SARS-CoV2	Confirms the existence of SARS-CoV2	Infection possible
Availability for Point-of-care	Yes	Yes	No	Yes
Application	entirely infection/immunity in a population	Individual testing	Individual testing	Triage to distinguish candidate for additional testing

#### 1.12.1 Clinical diagnosis

COVID-19 can show different signs and symptoms. Some people might not have any symptoms at all, while others could get very sick with serious lung problems or even die. Many people have been found to have infections without showing any symptoms, but some of these patients may later develop long-lasting health problems. We don't know the exact percentage of people who are infected but don't show symptoms. However, some reports say that as many as 80 % of infections might not cause noticeable signs of illness. In cases where there might be a medical problem, doctors look at various health factors. These include the patient's health history, age, gender, any other health issues, symptoms like dry cough, fever, sore throat, trouble breathing, mucus production, tiredness, muscle or joint pain, diarrhea, smell and taste ability, nausea or vomiting, loss of appetite, headaches, and chills.

##### 1.12.1.1 Radiographic testing

It's one of the ways doctors check for health issues after seeing breathing problems. It is used early in a disease and includes tests like chest CT scans, chest X-rays, or lung ultrasounds. X-rays are not very good at detecting the early stages of the disease, but they can be useful for COVID-19 patients in the intensive care unit. Using different X-ray tests along with RT-PCR in samples from infections could help improve the accuracy of RT-PCR tests and reduce false results. The results of these methods showed that most COVID-19 patients had problems in the lower part of both lungs. Lung cavitation, pneumothorax, and pleural effusions don't happen very often [[102], [103], [104]].

#### 1.12.2 Laboratorial diagnosis

##### 1.12.2.1 Serological test

A blood test checks if your blood has certain antibodies for the SARS-CoV-2 coronavirus. The result shows that you have the infection. Antibodies help protect the body. The body makes them when it gets infected with a virus. It will take 14–21 days for your body to make the right antibodies and release enough of them into your blood so that they can be detected. Blood tests help find viruses linked to past illnesses that can be used for treatment. To find out if the body is reacting to the viral spike (S) protein, doctors check for IgG or IgM antibodies using a test called ELISA. To check for a COVID-19 infection, a test is made that either looks for something similar to the virus or uses an antibody that catches the virus. IgM antibodies show up a few days to a week after infection, and IgG antibodies appear after about 8 days. Serology testing is not useful for diagnosing a current SARS-CoV-2 infection and should only be used in studies that track disease patterns [[105], [106], [107]]. Some patients also had higher levels of IL-6, C-reactive protein, cardiac troponin, ferritin, and myoglobin [108].

##### 1.12.2.2 Investigation of proinflammatory cytokines

In COVID-19 patients, there were too much of certain proteins called cytokines, including IL-1β, IL-1RA, IL-7, IL-8, IL-9, IL-10, basic FGF, G-CSF, GM-CSF, IFN-γ, IP-10, MCP-1, MIP-1A, MIP-1B, PDGF, TNF-α, and VEGF [109].

##### 1.12.2.3 RT–PCR

Nucleic acid tests that use RT-PCR are better at finding viruses than the current blood tests. They are more sensitive and accurate. Samples from breathing tubes, blood, and mucus are used in this test. The first step is to separate the viral RNA and change it into cDNA. Taq DNA polymerase is used to make more copies of the cDNA. Molecular methods are very important for diagnosing COVID-19 because of urgent situations, the high costs of traditional testing, and the need for new ways to test quickly [110]. PCR, which can copy DNA from entire groups of organisms, was used to identify viruses before the introduction of RT-PCR. However, because it is hard to sequence many colonies at once, it couldn't identify a growing number of samples [103]. Even though their genes are very similar, the genes that make the envelope, nucleocapsid, RNA-dependent RNA polymerase proteins, and spike are important for testing SARS-CoV-2 using RT-qPCR. Most tests look for two parts of the virus's genetic material. However, if there aren't enough testing supplies, it might be okay to use just one set of primers, but this should be carefully checked in each lab first [111,112]. SARS-CoV-2, like SARS-CoV, uses the ACE2 receptor to enter the cell. The ACE2 receptors play a key role in how infections develop in body tissues. The complete genetic material from infected patients was very similar to each other. The ACE2 proteins acted as a doorway for the SARS-COV-2 virus. SARS-Cov-1 was diagnosed using a test called RT-PCR within 5 days after the infection started. Both SARS-CoV-1 and the MERS virus have been shown to use this type [113]. If there aren't enough kits to extract viral genetic material, you can heat the sample first instead of extracting RNA [114]. Changes in the viral RNA sequence will affect RT-PCR results. Also, because viruses change over time, it's possible to get wrong results that say someone doesn't have the virus when they do [115]. Loop-mediated isothermal amplification and CRISPR tests, along with other molecular technologies, have been used for diagnosing diseases [116,117].

### 1.13 Treatment of COVID-19 virus

#### 1.13.1 General drug therapy

There is presently no proven treatment for COVID-19; however, various experiments are being performed to identify and test new and repurposed medications, and vaccines. The immune response or the pathophysiology of the virus are the targets of investigational drugs. Ribavirin as a nucleoside analog was one of the primary drugs examined for COVID-19 treatment [118]. Remdesivir is another nucleoside analog used for SARS-CoV, COVID-19, and MERS-CoV therapy [119]. HIV protease inhibitors lopinavir and ritonavir bind to SARS-CoV-2 endopeptidase C30 to lessen respiratory harm [120]. The other HIV protease inhibitor that suppresses SARS-Cov-2 viral antigen replication expression is nelfinavir [121]. Lopinavir/ritonavir, ribavirin, and IFN-β combination through blocking viral RNA are more effective than lopinavir-ritonavir [122]. IL-6 serum levels were increased between COVID-19 patients and humanized monoclonal anti-IL-6 receptor tocilizumab administration was efficient Feel secure when caring with COVID-19 patients [123]. By destroying ACE2, chloroquine as a malaria prevention drug has shown to be efficient in repressing SARS-CoV-2. Remarkably, it is significant as an inexpensive medication to treat COVID-19 [124]. Hydroxychloroquine participates in inhibiting SARS-CoV-2 by repressing virus repetition and inflammatory responses. Some studies showed that the combined effect of hydroxychloroquine with azithromycin is greater in eliminating the virus. Baricitini regulates endocytosis by binding to AP2-related protein kinase 1 and repress replication of SARS-CoV-2 [125]. Some evidence recommended that a combination of baricitinib beside lopinavir-ritonavir is useful when treating COVID-19 [126]. Children and adults alike tolerate arbidol as an antiviral medication that inhibits the SARS-CoV-2 and COVID-19 viral replication pathway [127]. Evaluation of Favipiravir vs umifenovir in COVID-19 determined that favipiravir with inhibition of viral RNA polymerase is more effective than umifenovir [128]. Ciclesonide, as a corticosteroid prescribed to treat allergies, is another drug that has significant antiviral properties against COVID-19, MERS-CoV-2, and SARS-CoV-2 [129]. Monoclonal antibody therapy is a significant immunotherapy for treating viral infections in specific people.

#### 1.13.2 Cell therapy

Cell therapy is utilized in the management of many viral infections, including COVID-19 [130]. It is possible to keep immune-modulating stem cells, particularly mesenchymal stem cells (MSCs), for possible use in medicine [131]. Hematopoietic stem cells (HSCs), mesenchymal stem/stromal cells (MSCs), pluripotent stem cells (PSCs), and fetal tissue-derived stem cells are among the different kinds of stem cells. These cells could be helpful in cell therapy because of their capacity to differentiate, regenerate, and create cytokines [132,133]. MSCs have an important impact on the regulation of inflammatory progression as well as the regeneration of compromised cells. Besides, IL-1 and TNF stimulation of MSCs will alter the immunosuppressive mechanism. MSCs regulate the inflammatory process by regulating the immune system. Chemokines, cytokines, exosomes, and growth factors can alter the function of MSCs [134]. The most important advantage of treatment with these cells is their isolation from a range of tissues including fat, oral tissue, bone marrow, blood, menstrual blood, placenta, umbilical cord, membrane, and amniotic fluid. Rapid growth in the shortest time and the ability of these cells to self-renew are some of their advantages [135,136]. It is believed that MSCs can control the coronavirus-induced cell-mediated inflammatory response and lessen acute lung injury. Additionally, MSCs are impervious to contamination and absence of the ACE2 protein, which is necessary for coronavirus invasion of cells. As a result, MSCs will increase the number of lymphocytes and regulatory DCs, boosting their antiviral properties, and resulting in lower IL-6, CRP, IL-8, and TNF levels in COVID-19 patients. During the initial phase of infection, most COVID-19 patients have no notable clinical symptoms. Mild or high fever, cough, sore throat, muscle discomfort, and body pain are all common symptoms. In certain people, shortness of breath can lead to a severe deterioration in health in the later stages of the illness. Since inflammation causes the release of inflammatory cytokines that overwhelm the respiratory system, including granulocyte colony-stimulating factor, IL2, IL6, MCP1, IP10, TNF, and MIP1A, immune system deficiencies are the primary cause of death for patients in severe cases. The immune system in COVID-19 is unable to switch off and releases a disproportionate amount of cytokines, creating an unfavorable atmosphere for the infection. This cytokine storm's uncontrolled inflammation compromises pulmonary capacity, causing patients to experience trouble breathing and ultimately perish. Organ loss, edema, secondary infection, heart injury, and ARDS may all result from the cytokine storm. MSCs are believed to help stabilize the immune system and prevent it from being overactive. The immune system must be balanced in this way since if it is completely shut down, patients' ability to resist infections will be compromised [137,138]. Anecdotal reports of anti-IL6 antibody monoclonal antibodies rescuing patients demonstrate the potential for controlling the COVID-19-induced cytokine storm during infection. MSCs have potent anti-inflammatory and immunomodulatory properties, allowing them to control cytokine storms by enhancing endogenous tissue repair and inhibiting immune system overactivation. MSCs have unique immunoregulatory properties that enable them to control the activities of a variety of immune cells [139,140]. The field of cell-based therapeutics has expanded due to the capacity to accurately edit the human genome using palindromic repeats like CRISPR and endonucleases like zinc finger nucleases. This has paved the way for more effective and individualized next-generation therapies. Following entry into infected cells, the SARS-CoV-2 RNA genome may be screened or rendered inactive. One of the most promising candidates for the action of CRISPR/Cas13 within infected cells has been found to be ORF1a/b. Therefore, a more tailored and responsive therapeutic strategy for viral diseases like SARS-CoV-2 may be developed using CRISPR technology. Therapeutic medications and improved therapeutic agents will be delivered by next-generation stem cell-based treatments on their own. As a result, by recruiting gene-editing platforms, the modern generation will edit disease-causing mutations or reduce the immunogenicity of allogeneic cells. MSCs can exert their immunomodulatory effects through a variety of mechanisms, according to research. MSCs are thought to facilitate lung regeneration by engraftment and transdifferentiation in the past. On the other hand, backed up MSCs' paracrine function in lung regeneration. MSCs seem to be promising interviewees for the treatment of a variety of illnesses, including ARDS, but questions about their efficacy, cell viability, and regulatory problems have been raised about their use in patients [141,142]. Cell-based treatment has emerged as a viable treatment sector for the treatment of terminal diseases such as diabetes, heart disease, ARDS, neurodegenerative disorders, liver damage, muscle degenerative problems, metabolic problems, sepsis, hematopoietic and immune system disturb and malignancies. Numerous studies have suggested the resistance Generation murine polyomavirus from pluripotent undifferentiated cells inflammation, with relation to embryonic and adult stem cells, against a variety of virus infections such as HIV1 and HIV2, cytomegalovirus, liver disease caused by the hepatitis B virus, either acute or chronic, Retrovirus, Myxoma virus [143,144]. It was found that pluripotent and multipotent stem cells are protected from viral infection by the intrinsic synthesis of interferon-stimulating genes (ISG). It was also noted that the interferon-induced transmembrane (IFITM) family of proteins provides this protective mechanism to the ISG of stem cells [145,146]. However cell therapy would not be a way of eradicating or curing SARS-CoV-2, several proofs of research promoted the idea that affected patients could be better able to resist and endure the infection. The distinctive characteristics of stem cells, including their potential for renewal and regeneration, play a crucial role in diverse therapeutic results. These qualities encompass the attenuation of inflammation, release of cytoprotective substances, mitochondrial transfer, diminution of apoptosis, antioxidant activity, and augmentation of the immunological response. Moreover, there is evident evidence that stem cells protect against influenza virus infection by facilitating the restoration of lung damage. Researchers have focused a lot of emphasis on mesenchymal stem cells (MSCs) because of their potential as cell-based treatments for COVID-19. Their regenerative, directional, anti-inflammatory, and immunomodulatory qualities are largely responsible for this interest. An extremely sick COVID-19 patient's pneumonia significantly improved after receiving an intravenous/intratracheal infusion of human umbilical cord mesenchymal stem cells (hUC-MSCs), and the patient was released from the intensive care unit within a week. There were no negative consequences linked to hUC-MSCs. Serum bilirubin, C-reactive protein (CRP), aspartate aminotransferase (AST), and alanine aminotransferase (ALT) levels all significantly decreased after the initial infusion. Three days after the initial injection, the lymphocyte levels rose to normal ranges and the white blood cell (WBC) and neutrophil counts returned to normal. Reduction of inflammation and healing of antiviral immune cells and impacted organs were made possible by the infusion of hUC-MSCs. Furthermore, by producing their target proteins, it is hypothesized that hUC-MSCs may have localized to repair damaged tissues and neutralize inflammatory cytokines like interleukin-6 (IL-6) and granulocyte colony-stimulating factor (G-CSF) [147].

##### 1.13.2.1 The role of mesenchymal stem cells in COVID-19-associated complications

Acute respiratory distress syndrome (ARDS) is one of the most common COVID-19 complications. ARDS is characterized by sudden respiratory failure and protein-rich pulmonary edema, which are produced by severe inflammation and damage to the lung tissue and epithelium [148]. The COVID-19 treatment plan is critical, and it includes strategies such as hydration management and lung-protective ventilation. There is presently no established solution for acute respiratory distress syndrome (ARDS), which significantly increases morbidity and fatality [149]. One of the main characteristics of the pathophysiology of acute respiratory distress syndrome (ARDS) is the imbalance between pro-inflammatory and anti-inflammatory molecules. Regulating these mediators, particularly cytokines, is therefore a feasible therapeutic strategy. Mesenchymal stem cells (MSCs) have demonstrated therapeutic effects for ARDS in multiple trials due to their unique immunomodulatory capabilities. Studies have demonstrated that MSCs reduce lung permeability, enhance alveolar fluid clearance, combat infections, and modify inflammatory responses in ARDS. A range of soluble paracrine compounds, including anti-inflammatory cytokines like interleukin-10 (IL-10) and interleukin-1 receptor antagonist (IL-1RA), can also be secreted by MSCs. According to one study, serum from patients with acute respiratory distress syndrome (ARDS) activated human bone marrow-derived mesenchymal stem cells (BMMSCs). Their elevated levels demonstrated that these MSCs were more successful in lowering respiratory disease as a result of their activation. This effect was associated with improved therapeutic potential and increased expression of interleukin-10 (IL-10) and interleukin-1 receptor antagonist (IL-1RA). This was demonstrated by pulmonary edema, a decrease in lung damage scores, and the accumulation of inflammatory cells and cytokines in bronchoalveolar lavage fluid [97]. Mesenchymal stem cells (BMMSCs) were activated following preconditioning with serum from patients with acute respiratory distress syndrome (ARDS). Reactivated MSCs produced increased interleukin-1 receptor antagonists (IL-1RA) and interleukin-10 (IL-10) while suppressing the release of pro-inflammatory cytokines. A robust immunomodulatory response characterized by elevated prostaglandin E2 (PGE2) secretion was facilitated by the upregulation of vascular endothelial growth factor (VEGF) and toll-like receptor 4 (TLR-4) gene expression. When administered to ARDS porcine models, these allogenic MSCs significantly reduced the levels of pro-inflammatory cytokines and enhanced the production of anti-inflammatory mediators. Consequently, culturing allogenic MSCs in a patient-like environment prior to treatment may activate them and have more immunomodulatory effects than infusing non-activated MSCs. Acute respiratory distress syndrome (ARDS), respiratory failure, and severe pneumonia are the hallmarks of the H7N9 influenza virus infection. In a clinical study, individuals with acute respiratory distress syndrome (ARDS) caused by H7N9 were administered mesenchymal stem cells (MSCs). The control group consisted of patients with H7N9-induced ARDS who did not receive MSC transplantation. The study's findings demonstrated that individuals treated with allogenic MSCs derived from menstrual blood had a much higher survival rate than the control group [150]. Adipose tissue is a safe and dependable source of mesenchymal stem cells (MSCs). Adipose-derived stem cells (ASCs) are notable for their potent anti-inflammatory properties and can be obtained by a minimally invasive aspiration technique. These stem cells release chemicals that promote angiogenesis and accelerate the proliferation of vascular endothelial cells, including vascular endothelial growth factor (VEGF) and platelet-derived growth factor (PDGF) [151,152]. They possess the capacity to enhance the respiratory function of COVID-19 patients, alongside their immunosuppressive action, which induces the production of TGF-1, HGF, and INF-y [153]. After a 5-year follow-up period, the researchers also discovered that MSC implantation had no adverse effects on the human body. MSC transplantation significantly raised the survival rate of patients with H7N9-induced ARDS in both preclinical and clinical studies [150]. Given that coronaviruses and H7N9 influenza have similar outcomes, such as the development of acute respiratory distress syndrome (ARDS) and subsequent multi-organ failure, mesenchymal stem cell (MSC)-based therapy may be a potential approach for COVID-19 prevention.

##### 1.13.2.2 Exosomes' role in COVID-19

The majority of research on mesenchymal stem cell (MSC)-derived exosomes has shown that they have immune-modulatory, anti-inflammatory, and regenerative capabilities that are on par with those of the parent MSCs. Exosomes made from mesenchymal stem cells (MSCs) have shown promise in clinical trials as an acellular substitute for cell-based treatments for acute respiratory distress syndrome (ARDS). The results showed that the cytokine storm and pro-inflammatory signaling molecules that are mostly in charge of the pathophysiology of ARDS were significantly reduced after these exosomes were infused. Additional investigation revealed that exosomes raised anti-inflammatory signaling mediator levels, which may lessen lung damage by improving the alveolar epithelium's permeability and advantageous characteristics [154]. As a result, contact with oxygen-rich air is made easier. Exosomes' ability to transport mitochondria to alveolar cells has been shown to improve cell survival and encourage cellular regeneration. These discoveries have opened the door for this novel acellular substitute to be used therapeutically. MSC-derived exosomes have been shown to directly inhibit viral replication in addition to their effects in preclinical models of acute respiratory disorders. Essential components of exosomes, microRNAs (miRNAs) are known to have a major role in several physiological processes, such as immunological modulation, development, and epigenetic control. Near-infrared dyes have been used in numerous studies to track the in vivo biodistribution of exosomes after systemic treatment in a range of animal models. Exosomes have been shown in several studies to target various organs, including the brain, as demonstrated by intracerebral hemorrhagic rat models. In a mouse model of acute renal failure, systemically injected exosomes accumulated in the kidneys, revealing their robust paracrine routes and ability to rapidly target inflammatory regions. Furthermore, exosome-secreted miRNAs are important for accelerating lung regeneration, notably in people with viral infections like influenza, ventilator-associated lung injury, and hypoxia-induced pulmonary hypertension [155]. Certain miRNAs, including let-7, miR-21, miR-290, and miR-200, are crucial for lung regeneration, immunological regulation, and immune control when they are modulated in animal models during the early and late phases of lung injury recovery. Stem cell-derived exosomes can reduce inflammation and hypertension by blocking hypoxia-related signaling pathways, which is particularly advantageous in lung disorders. Moreover, it has been demonstrated that exosomes generated from MSCs directly inhibit viral multiplication.

## 2 Conclusion

Globally, the COVID-19 pandemic is having an immediate and significant impact on day-to-day living. Nowadays, carrying out professional duties effectively requires a thorough understanding of SARS-CoV-2 transmission, preventative techniques, and screening procedures. People who carry COVID-19 may show up as asymptomatic carriers or develop a serious sickness that includes sepsis and abrupt respiratory failure. About 20 % of hospitalized patients and 5 % of COVID-19 cases have severe symptoms that necessitate critical care. Furthermore, oxygen therapy is necessary for more than 75 % of hospitalized COVID-19 patients. One of the main goals of treating COVID-19 is to effectively manage acute respiratory distress syndrome (ARDS) and acute hypoxemic respiratory failure (AHRF). According to preliminary findings, dexamethasone medication lowers the 28-day death rate for individuals needing additional oxygen from 15 to 11 days when compared to standard care. Current research is testing anticoagulants, immune modulators, and antiviral medications. Before an effective vaccine is developed, the fundamental measures for reducing dissemination include touch tracing, face masks, and social distancing. It may be able to use additional prophylactic measures like hyperimmune globulin and monoclonal antibodies. In addition to clinical and public health activities, COVID-19 prevention and supervision strategies would require clinical research and molecular genetics. In this way, numerous clinical trials for MSC therapy have been conducted. The evidence suggests that MSC therapy not only significantly reduces damage but also aids in the healing process by fostering immunological tolerance and protection. In this regard, MSC therapies offer COVID-19 patients a promising but difficult alternative. The main objective of this research is to provide a comprehensive review of the translational and cellular pathobiology of COVID-19, combining clinical, molecular, and immunological aspects, with a particular focus on novel therapeutic strategies such as mesenchymal stem cell (MSC) therapy. These therapies appear to be the best options for reducing inflammation, promoting tissue repair, preventing permanent damage, and reducing death. To evaluate the effectiveness of MSC treatment and its related functions, phase-3 trials are also required.

This review not only synthesizes molecular, clinical, and immunological dimensions of COVID-19 but also introduces a translational viewpoint by highlighting innovative therapeutic strategies, especially mesenchymal stem cell (MSC) therapy, which remains an underexplored but promising area. By integrating current evidence on pathobiology with evolving treatment paradigms, this work provides a roadmap for developing more effective and personalized interventions. Furthermore, it underscores the necessity of multidisciplinary approaches—spanning virology, immunology, epigenetics, and regenerative medicine—to comprehensively address current and future pandemic challenges. Such an integrated strategy is essential for tailoring novel diagnostic and therapeutic protocols, particularly for severe COVID-19 manifestations like cytokine storms and ARDS.

Compared to existing review articles that often focus solely on virological or clinical dimensions of COVID-19, the present work offers a more integrated approach by linking molecular insights with clinical strategies and emerging therapeutic innovations. While other studies have addressed mesenchymal stem cell therapy in isolated contexts, this review uniquely situates MSCs within a broader framework of immunomodulation, epigenetic regulation, and regenerative intervention. This comparative perspective underscores the originality and translational value of our work, particularly in highlighting MSC therapy not merely as an adjunct, but as a potentially central approach for managing severe COVID-19 pathology.

## CRediT authorship contribution statement

**Ali Akbar Samadani:** Writing – review & editing, Validation, Methodology, Investigation, Conceptualization. **Sogand Vahidi:** Writing – original draft, Investigation. **Kosar Babaei:** Writing – original draft, Methodology. **Seyedeh Elham Norollahi:** Writing – original draft, Investigation. **Kourosh Delpasand:** Writing – original draft, Resources. **Elaheh Asghari Gharakhyli:** Writing – original draft, Investigation.

## Declaration of competing interest

Hope you are doing well. According to the submission of our manuscript which is entitled: “Comprehensive and translational pathobiology of COVID-19 based on cellular and molecular techniques” In this journal, all authors declare that there is no conflict of interest and also all the ethical standards considered carefully. Remarkably, all the authors studied and confirmed the final edited version of this manuscript.

We hope that the manuscript will receive a fair review and will hear from you positively soon.

## Data Availability

The data that has been used is confidential.
