# Supplementary material for: Comprehensive and translational pathobiology of COVID-19 based on cellular and molecular techniques
Source: Pract Lab Med. 2025 Aug 11;46:e00497. doi: 10.1016/j.plabm.2025.e00497 (PMC12391832; doi:10.1016/j.plabm.2025.e00497)
Supplement: Multimedia component 1 [file mmc1.docx]

**Dear Editor,**

Hope you are doing well. By the way, I submitted this manuscript which is entitled: **Comprehensive and translational pathobiology of COVID-19 based on cellular and molecular techniques** in this journal. Importantly, due to the serious sanctions of the USA, we are not able to transfer the money. and request a full waiver for APC. I would be so grateful if you would consider my request. Thank you so much for your deep understanding and attention.

With best and warm regards

**Ali Akbar Samadani, PhD,** Guilan Road Trauma Research Center, Trauma Institute, Guilan University of Medical Sciences, Rasht, Iran

Email:a.a.hormoz@gmail.com
